# Antidepressant treatment in inflammatory bowel disease: a systematic review and meta-analysis

**DOI:** 10.1097/MEG.0000000000002768

**Published:** 2024-04-29

**Authors:** Frances Weston, Ben Carter, Nick Powell, Allan H. Young, Calum D. Moulton

**Affiliations:** a Centre for Affective Disorders; b Department of Biostatistics, Institute of Psychiatry, Psychology and Neuroscience, King’s College London; c Department of Digestion, Metabolism and Reproduction, Imperial College London; d National Affective Disorders Service, South London and Maudsley NHS Foundation Trust; e Division of Psychiatry, Department of Brain Sciences, Imperial College; f Psychological Medicine Unit, St Mark’s Hospital, London, UK

**Keywords:** antidepressants, depression, inflammatory bowel disease, meta-analysis, systematic review

## Abstract

Around 25% of patients with inflammatory bowel disease (IBD) have depressive symptoms, yet antidepressants have been poorly studied in IBD. We systematically searched IBD studies testing antidepressants in four databases. Outcomes were depressive symptoms, anxiety, IBD disease activity, quality of life (QoL) and adverse events. For randomized controlled trials (RCTs), we performed random-effects meta-analysis of the standardized mean difference (SMD) in posttreatment scores between antidepressant and placebo groups. Risk of bias was assessed using the Cochrane Common Mental Disorders Depression Anxiety and Neurosis Group tool (clinical trials) and Newcastle–Ottawa scale (cohort studies). We included 11 studies (*n* = 327): three placebo-controlled RCTs, two nonrandomized trials, and six other study types. In the pooled analysis, antidepressants improved depressive symptoms [SMD = −0.71 (95% confidence interval (CI) −1.32 to −0.10), *P* = 0.02, *I*^2^ = 51%] and QoL [SMD = 0.88 (95% CI 0.30–1.45), *P* = 0.003, *I*^2^ = 44%] more than placebo. Serotonin and noradrenaline reuptake inhibitors (SNRIs) alone improved depressive symptoms [SMD = −0.95 (95% CI −1.45 to −0.45, *P* < 0.001, *I*^2^ = 11%], anxiety [SMD = −0.92 (95% CI 1.72 to −0.13), *P* = 0.023, *I*^2^ = 65%] and QoL [SMD = 1.14 (95% CI 0.66–1.62), *P* < 0.001, *I*^2^ = 0%]. The three RCTs were of good quality. In conclusion, based on three small but good-quality studies, antidepressants improve depressive symptoms and QoL compared to placebo in IBD. SNRI antidepressants may also improve anxiety. A fully powered study of antidepressants in IBD is needed.

## Introduction

Inflammatory bowel disease (IBD) is a debilitating, relapsing-remitting illness, which primarily comprises ulcerative colitis and Crohn’s disease. IBD is associated with unpredictable, painful and often disabling symptoms for patients [1]. Rates of depression are over twice as high in individuals with IBD than in the general population, and these rates are significantly higher for those with active IBD compared to inactive IBD [2]. Notably, the comorbidity of depression is associated with poor IBD outcomes, including increased risk of IBD relapse, hospitalisation, requirement for biologic therapy and surgery [3]. This suggests that effective treatment of depression could improve both quality of life and IBD outcomes.

Antidepressants are an effective treatment for patients with depression and anxiety in the general population and are also commonly used to treat psychological comorbidities in patients with IBD [4]. The comorbidity of IBD, however, poses potential barriers to effectiveness and tolerability of antidepressants. These challenges include the burden of IBD symptoms (e.g. pain and diarrhoea), impaired drug absorption [5], and possible increased vulnerability to side-effects in this population [4]. A systematic review of antidepressants in IBD from 2017 was limited by lack of available trials and lack of data on adverse events [6]. A recent meta-analysis found antidepressants to be effective in patients with IBD. The findings of this synthesis, however, were based primarily on studies where randomisation or blinding procedures were not reported [7], thereby seriously undermining the validity of the findings. Furthermore, the authors did not analyse by antidepressant class, meaning it remains unclear which specific antidepressants are likely to be most beneficial in patients with IBD.

We therefore performed an updated systematic review and meta-analysis of the effect of antidepressants in patients with IBD, in which we only meta-analysed randomized controlled trials (RCTs). We hypothesized that antidepressants would improve depressive symptoms, anxiety symptoms and quality of life in IBD.

## Methods

The writing of this systematic review was guided by the standards of the Preferred Reporting Items for Systematic Review and Meta-Analysis (PRISMA) Statement [8], in which studies that meet review criteria are examined and RCTs and with sufficient data pooled for meta-analysis.

### Study selection

We included studies that met all the following criteria: (a) recruited participants clinically diagnosed with any type of IBD (Crohn’s disease, ulcerative colitis or indeterminate colitis); (b) were RCTs, non‐RCTs, case-control studies, prospective and retrospective observational studies, case series or case reports; (c) participants were prescribed any of the following antidepressants at the start of the research: a selective serotonin reuptake inhibitor (SSRI) (sertraline, fluoxetine, paroxetine, fluvoxamine, citalopram, escitalopram), tricyclic antidepressant (amitriptyline, nortriptyline, imipramine, clomipramine, desipramine), tetracyclic antidepressant (mirtazapine, mianserin), serotonin and noradrenaline reuptake inhibitor (SNRI) (venlafaxine, duloxetine), monoamine oxidase inhibitor (moclobemide, phenelzine, selegiline) or other antidepressant (bupropion, vortioxetine, vilazodone, trazodone) and (d) depression was assessed before and after treatment. Studies were excluded if they met the following criteria: (a) study was qualitative only; (b) study recruited a paediatric population (<18 years) and (c) study was a review article presenting no original data.

### Study extraction

We systematically searched PubMed, Cochrane Library Trials, Embase (via Ovid) and Web of Science for studies published from inception to 31 December 2023. The full search strategy is included as supplementary material, Supplemental digital content 1, http://links.lww.com/EJGH/B20. To identify unpublished or ongoing studies, we searched trial registries including ClinicalTrials.gov and the European Union (EU) clinical trials register. A manual search of references was carried out on the included studies and four reviews in the area of antidepressant use in IBD.

Two authors (F.W. and C.D.M.) independently performed the literature search and resolved differences over inclusion through discussions and consensus. Two reviewers independently screened titles and abstracts for potentially eligible studies and assessed them for inclusion.

From abstracts fulfilling inclusion criteria, full-text articles were reviewed, and data extraction performed for studies still meeting inclusion criteria.

### Data extraction

For each study, we extracted the following data: author, year, country, study design, patient age and sex distribution, IBD type, IBD duration, baseline IBD activity [faecal calprotectin, validated disease indices such as the Crohn’s Disease Activity Index (CDAI), serum C-reactive protein], baseline depression and anxiety status, antidepressant treatment (medication, dose, duration of treatment), depression and anxiety after treatment, changes in IBD symptoms or disease activity scores, and frequency of incident adverse effects.

### Risk of bias assessment

Due to the various study designs, two quality assessment methods were used. For RCTs and non-RCTs, we used the Cochrane Common Mental Disorders Depression Anxiety and Neurosis group (CCDAN) tool, as it has been specifically designed to assess trials related to depressive disorders [9]. The CCDAN consists of 23 items, and gives a score out of 46, with a score of 20 or more suggesting high quality. For cohort studies, the Newcastle–Ottawa scale (NOS) was used, which gives studies a score out of 8, with a higher score signifying lower risk of bias [10].

### Outcomes

The primary outcome was any validated measure of depressive symptoms at the latest time-point within the study. Secondary outcomes were anxiety symptoms, quality of life (QoL), IBD disease activity and adverse events using any validated instrument.

### Measures of effect

For each study, effect size estimates were calculated using the standardized mean difference (SMD) in depressive symptoms after treatment. Where not provided directly, the standard error of each group sizes was estimated [11].

### Statistical analysis

We considered pooling with a minimum of three RCTs that were clinically homogeneous. Studies were weighted using an inverse-variance method, where studies with larger precision are afforded greater weight. Pooled effect estimates were calculated using a random-effects model [12]. Analyses were performed using STATA 17.0.

### Heterogeneity

Heterogeneity between studies was quantified by calculating the *I*^2^-statistic, *I*^2^ between 25, 50 and 75% suggesting low, moderate and high heterogeneity, respectively [13]. Moderate and high heterogeneity were explored using subgroup analysis by antidepressant class. We did not assess for publication bias due to the small number of studies in the meta-analyses.

## Results

**Fig. 1.**
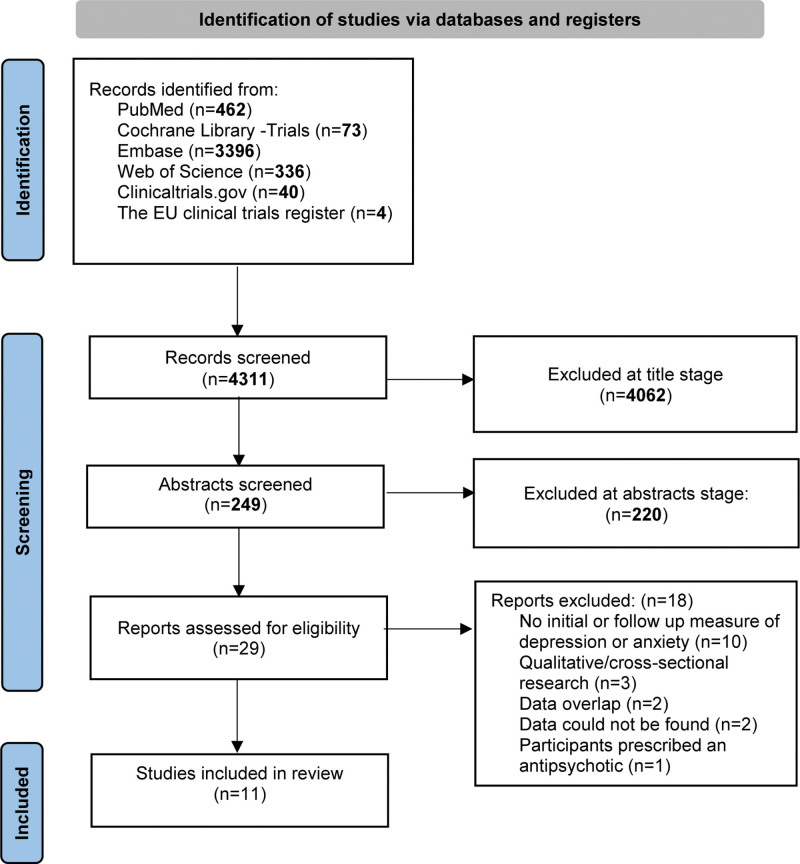
PRISMA flow diagram outlining the systematic review stages.

A total of 4311 studies were retrieved and screened for relevance, of which 4282 papers were excluded at title/abstract stage and 29 assessed for eligibility. Following full-text review, 11 studies were included (Fig. 1). Table 1 shows the characteristics of included studies.

**Table 1.** Characteristics of included studies

Author (year), country	Study design	Sample size, sex, mean age (SD)	IBD type; mean duration (SD)	IBD baseline measures and mean score (SD)	IBD medication	Baseline depression and anxiety measure: mean score (SD)	Antidepressant type, dose, and duration
Daghaghzadeh *et al*. (2015), Iran	Placebo-controlled RCT	*N* = 35: 19 female, 16 male. 38 years (8.2)	Ulcerative colitis: 22, Crohn’s disease: 13; 6.49 years (3.27)	LCAI for both ulcerative colitis and Crohn’s disease 6.88 (3.8) with no flair in prior 6 months.	Both groups given mesalazine 2–4 g/day	HADS-D 9.22 (3.45). HADS-A 8.17 (4.29).	Intervention: duloxetine 30 mg/day for 1 week, then 60 mg/day for next 11 weeks. Placebo: same form and packaging as intervention.
Mikocka-Walus *et al*. (2017), Australia	Placebo-controlled RCT	*N* = 26: 12 female, 14 male. 37.4 years (13.2)	All Crohn’s disease: intervention: 14.98 years (13.1), placebo: 12.21 years (8.1)	CDAI: intervention: 63.8 (44.4), placebo: 66.4 (44.7), faecal calprotectin: intervention: 46.4 (33.2), placebo: 98.1 (94.4). All in remission but flare in last 12 months.	Remained on current Crohn’s disease medication: complementary (*n* = 12), mesalazine (*n* = 7), biologics (*n* = 13), immunomodulators (*n* = 19), prednisolone (*n* = 1). Medication unchanged throughout.	HADS-D: intervention: 3.9 (2.9), placebo: 3.6 (3.1). HADS-A: intervention: 5.3 (4.1), placebo: 4.9 (3.4)	Intervention: fluoxetine 20 mg/day. Placebo: gelatine capsules filled with microcrystalline cellulose
Liang *et al*. (2022), China	Placebo-controlled RCT	*N* = 45: 20 female, 25 male. 40 years (13.12)	Ulcerative colitis: 22 years, Crohn’s disease: 23 years	Mayo score for ulcerative colitis: 5.80 (3.81). CDAI for Crohn’s disease: 268.02 (119.8).	Remained on current IBD medication: mesalazine (*n* = 14), corticosteroids (*n* = 3), immunomodulators (*n* = 12), biologics (*n* = 16)	HADS (MDD): intervention: 8.81, placebo: 8.81 HADS (GAD): intervention: 10.09, placebo: 10.09	Intervention: venlafaxine (sustained-release form) 75 mg/day for 1 week, then increased to 150 mg. Placebo: gelatine capsules filled with starch
Chojnacki *et al*. (2011), Poland	Nonrandomized controlled trial	*N* = 60: 39 female, 21 male. 30.6 years (8.8)	All ulcerative colitis, range 4–16 years	In remission for prior 6 months, confirmed endoscopically and using Mayo index.	Both groups given 2.0 g of aminosalicylates/day	BDI: 19.95 (4.49) HAMA: 20.35 (4.03)	Intervention: 2.0g aminosalicylates and tianeptine 37.5 mg/day for 12 months. Placebo: 2.0 g aminosalicylates and a placebo for 12 months
Hashash *et al*. (2022), USA	Open-label trial	*N* = 68: 41 female, 27 male. 23.8 years (4.8)	All Crohn’s disease (duration unknown)	HBI mild or no disease activity: 4.30 (5.0). At 4 weeks, Bupropion + BBTS-I: 4.7 (5.2), BBTS-I only: 3.7 (4.9).	Remained on current Crohn’s disease medications: aminosalicylates (*n* = 21), biologics (*n* = 35), steroids (*n* = 9), immunomodulators (*n* = 34)	HDRS at baseline: 12.62 (5.6). At 4 weeks -bupropion + BBTS-I: 12.7 (5.6), BBTS-I only: 10.7 (4.8). HARS at baseline: 13.86 (7.9). At 4 weeks, bupropion + BBTS-I: 14.6 (8.0), BBTS-I only: 9.6 (5.3)	Intervention: bupropion sustained release 100–300 mg/day for 8 weeks in patients continuing to experience fatigue after behavioural therapy alone (*n* = 33).
Yanartas *et al*. (2016), Turkey	Prospective cohort	N = 67: 43 female, 24 male. 40.71 years (12.71)	Ulcerative colitis: 36, Crohn’s disease: 31	CDAI group A: 197.41 (130.60), group B: 58.50 (74.94). MMS: group A: 2.71 (3.05), group B: 2.78 (3.42).	Remained on current IBD medication: mesalamine (*n* = 45), corticosteroids (*n* = 4), anti-TNF (*n* = 25), azathioprine (*n* = 29), methotrexate (*n* = 8), budesonide (*n* = 3)	HADS (MDD) 43.3%. Group A: 10.62 (3.61). Group B: 11.55 (2.85). HADS (GAD) 15%. Group A: 12.38 (4.38). Group B: 11.40 (4.60).	Sertraline 21.0%, escitalopram 15.8%, bupropion 12.3%, mirtazapine 12.3%, paroxetine 10.6%, venlafaxine 5.2%, duloxetine 3.5%, fluvoxamine 1.8% (sertraline + mirtazapine) 7.0%, (escitalopram + trazodone) 5.2%, (venlafaxine + mirtazapine) 1.8% and (sertraline + quetiapine) 3.5%.
Zanello *et al*. (2020), Italy	Retrospective cohort	*N* = 15: 9 females, 6 male. 48.27 years (13.7)	Ulcerative colitis: 6, Crohn’s disease: 9; 31.3 years (13.3)	CDAI or HBI for Crohn’s disease. Partial Mayo score for ulcerative colitis. In clinical remission.	Remained on current IBD medication: NSAIDs (*n* = 5), immunosuppressive (*n* = 1), TNF inhibitors (*n* = 5), polytherapy (*n* = 3)	HAM-D: 21.5 (7.4). HAM-A: 25.6 (8.1)	Sertraline (*n* = 10), citalopram (*n* = 1), paroxetine (*n* = 1), fluoxetine (*n* = 1), duloxetine (*n* = 2)
Walker *et al*. (1996), USA	Case Series	*N* = 8: 43.1 years (11.3)	IBD – type unknown	Structured gastrointestinal symptoms interview.	Not reported	HAM-D: 29.0 (7.8). Anxiety: N/A.	Paroxetine 20-40 mg OD according to response
Kast (1998), USA	Case report	*N* = 1: female, 33 years	Crohn’s disease, duration unknown	Ten watery bowel movements with severe abdominal cramping daily, dietary intolerance to all solid foods.	5 mg azathioprine, 60 mg prednisone, and 3 acetaminophen/oxycodone tablets daily. At 1 month, azathioprine and prednisone tapered off.	Anxiety-prominent major depressive episode	Phenelzine 45-90 mg/day according to response
Kast and Altschuler (2001), USA	Case report	*N* = 1: female, 44 years	Crohn’s disease, duration unknown	CDAI: 202. Pain throughout week. Bleeding and incontinence.	500 mg mesalamine 2×/day, tapered off after a few months	Major depression, superimposed on a chronic mild depressed state (dysthymia).	Fluoxetine 40 mg/day for depression changed to bupropion 150 mg twice a day, increased to 150 mg three times a day
Joshi and Dixit (2013), India	Case report	*N* = 1 male, 26 years	Ulcerative colitis, duration unknown	Urgency of defaecation, bloody diarrhoea and rectal pain. Past courses of steroids had minimal benefit.	Immunomodulators, and steroids in past. Past courses of steroids had minimal benefit.	Features of GAD	Mirtazapine 15 mg at night

BDI, Beck depression inventory; CDAI, Crohn’s disease activity index; GAD, generalized anxiety disorder; HADS, the hospital anxiety and depression scale; HAD-A, the hospital anxiety and depression scale – anxiety; HAD-D, the hospital anxiety and depression scale – depression; HBI, Harvey–Bradshaw index; HAMA, Hamilton anxiety scale; HARS, Hamilton anxiety rating scale; HAM-A, Hamilton rating scale for anxiety; HAM-D, Hamilton rating scale for depression; HDRS, Hamilton depression rating scale; IBD, inflammatory bowel disease; LCAI, Lichtiger colitis activity index; MMS, modified mayo score; MDD, major depressive disorder; RCT, randomized controlled trial; TNF, tumor necrosis factor.

### Randomized controlled trials

Three small placebo-controlled RCT’s were included. One used the SSRI fluoxetine for 12 months in 26 patients with Crohn’s disease in remission (but with a flare in the last year) [14]; one used the SNRI duloxetine for 6 months in patients with Crohn’s disease or ulcerative colitis in remission [15]; and the other used the SNRI venlafaxine for 6 months in 45 patients with ulcerative colitis or Crohn’s disease, which also included patients with active IBD [16]. For the duloxetine and fluoxetine studies, depression was not an inclusion criterion. For the venlafaxine study, participants required a score ≥8 on the Hospital Anxiety and Depression Scale (HADS), indicative of mild anxiety or depressive symptoms, though did not require a clinical diagnosis of depression.

Following treatment, the duloxetine and venlafaxine trials reported improvement in depression and anxiety scores compared to placebo. There was no significant change in depression or anxiety when using fluoxetine, though baseline depressive symptoms were low and depressive symptoms were a secondary outcome. For IBD control, the venlafaxine study found a significant decrease in both Mayo score and CDAI score after 6 months. The fluoxetine and control CDAI scores and faecal calprotectin levels were comparable at 12 months (Table 2).

**Table 2.** Summary of study results and outcomes for IBD papers

Author (year)	Follow-up duration	Changes in depression after treatment	Changes in anxiety and fatigue	Changes in IBD symptoms or control	Changes in quality of life	Significant adverse effects	Outcomes
Daghaghzadeh *et al*. (2015)	3 months	Greater decrease in depression with intervention vs placebo (*P* = 0.041)	Greater decrease in anxiety with intervention vs placebo (*P* = 0.049)	Greater decrease in severity of symptoms with intervention versus placebo (*P* = 0.02)	Greater increase in psychological WHOQOL score with intervention vs placebo (*P* = 0.038), using the WHOQOL-BREF. Sub-domain changes: physical (*P* = 0.001), social (*P* = 0.015), environmental (*P* = 0.26)	*n* = 1 stopped duloxetine due to SE’s. Nausea more frequent in duloxetine (*n* = 7) vs placebo (*n* = 2) (*P* = 0.049). Other side effects showed not different between duloxetine and placebo: dizziness (*n* = 4, *n* = 1), drowsiness (*n* = 3, *n* = 0), sexual side effects (*n* = 2, *n* = 0), insomnia (*n* = 2, *n* = 0)	1. Duloxetine significantly reduced depression and anxiety score in IBD patients. 2. Duloxetine also significantly decreased IBD symptom severity
Mikocka-Walus *et al*. (2017)	12 months; follow up at 3, 6, and 12 months	No significant group difference in depression score over 12 months (*P* = 0.96)	No significant group difference in anxiety score over 12 months (*P* = 0.98)	No significant difference between fluoxetine and control CDAI score (*P* = 0.98) or faecal calprotectin (*P* = 0.37) over 12 months. No difference in proportion in remission at any time point (*P* < 0.05)	No significant group difference in psychological WHOQOL score (*P* = 0.88). Sub-domain changes: physical QOL (*P* = 0.65), social relationships (*P* = 0.65) and environmental (*P* = 0.98)	*N* = 1 stopped due to side-effects of intervention. All side-effects resolved within first 2 weeks. Intervention SE’s: fatigue (*n* = 4), low mood/anxiety (*n* = 2); nausea, diarrhoea or vomiting (*n* = 2), dry mouth (*n* = 1), hot flushes (*n* = 1). Placebo AEs: muscle spasm (*n* = 2), nausea, diarrhoea or vomiting (*n* = 1).	1. Fluoxetine had no significant effect on depression or anxiety in Crohn’s disease. 2. Fluoxetine had no significant effect on Crohn’s disease activity.
Liang *et al*. (2022)	6 months; follow up at 3 and 6 months	Greater decrease in depression with intervention vs placebo at 3 months (*P* < 0.001) and 6 months (*P* < 0.001)	Greater decrease in anxiety with intervention vs to placebo at 3 months (*P* < 0.001) and 6 months (*P* < 0.001)	Ulcerative colitis group in intervention improved in Mayo score vs placebo at 6 months (*P* < 0.001). Crohn’s disease group improved in CDAI vs placebo at 6 months (*P* = 0.006)	Greater increase in IBDQ score in intervention vs placebo (*P* < 0.001)	Intervention: dizziness (*n* = 3), palpitation (*n* = 1), nausea (*n* = 2), and insomnia (*n* = 1). Placebo: nausea (*n* = 1) and diarrhoea (*n* = 1)	1. Venlafaxine significantly reduced depression and anxiety score in IBD patients 2. Venlafaxine also significantly reduced IBD disease activity after 6 months
Chojnacki *et al*. (2011)	12 months; follow up at 3, 6, 9 and 12 months	Greater decrease in depression with tianeptine vs placebo at 6 (*P* < 0.05), 9 (*P* < 0.01) and 12 months (*P* < 0.001)	Greater decrease in anxiety with tianeptine vs placebo after 9 (*P* < 0.001) and 12 months of treatment (*P* < 0.001)	MCDAI improved for tianeptine vs placebo (*P* < 0.01). No change in CRP for placebo between groups	Not reported	Intervention: nausea in week 1 (*n* = 4), and mild headaches (*n* = 3), none required discontinuation. Placebo: episodic headaches (*n* = 4)	1. Tianeptine significantly reduced depression and anxiety score in ulcerative colitis patients 2. Tianeptine also significantly decreased ulcerative colitis disease activity
Hashash *et al*. (2022)	3 months: latter 2 months with intervention	Significant decrease in depression in intervention group (*P* = 0.001). In the BBTS-I only group, significant decrease in depression (*P* = 0.02)	Significant decrease in anxiety following intervention (*P* = 0.008). In the BBTS-I only group, a significant decrease in anxiety (*P* = 0.02)	No significant change in HBI score in Bupropion + BBTS-I (*P* = 0.65) or BBTS-I only group (*P* = 0.39) over 8 weeks	Not reported	4 subjects in the bupropion group never took or stopped medication within 30 days because they felt that it was not needed (*n* = 2), made them feel anxious (*n* = 1) or felt no benefit (*n* = 1).	1. Bupropion significantly reduced depression and anxiety score in Crohn’s disease patients 2. Bupropion had no significant effect on Crohn’s disease activity.
Yanartas *et al*. (2016)	6 months	Greater improvement in depression in group adherent to antidepressant treatment vs patients nonadherent (*P* = 0.017)	Greater improvement in depression in group adherent to antidepressant treatment vs patients nonadherent (*P* = 0.001)	Significant decrease in mean CDAI score (SD) for antidepressant-adherent patients (*P* = 0.011) but no between-group differences	Greater increase in QOL SF-36 in group adherent to antidepressant treatment vs patients nonadherent to antidepressants (*P* = 0.010 to <0.001 for each QOL SF-36 subgroup)	1/3 experienced side-effects: drowsiness and fatigue (*n* = 5), sexual dysfunction (*n* = 4), weight gain (*n* = 4), insomnia (*n* = 3), anxiety (*n* = 2), nausea (*n* = 1).	1.Antidepressants significantly reduced depression and anxiety score in IBD patients 2. Antidepressants significantly reduced IBD activity
Zanello *et al*. (2020)	12 months; follow up at 1, 3, 6 and 12 months	Depression significantly decreased over 12 months (*P* = 0.001)	Anxiety significantly decreased over 12 months (*P* = 0.001)	Frequency of bowel movements significantly decreased over 12 months (*P* = 0.003). No significant change in abdominal pain or bleeding	Greater increase in QOL SF-36 in those with an anxiety or mood disorder in the ‘vitality’ domain (*P* = 0.03), ‘general health perception’ (*P* = 0.05) and ‘mental health’ (*P* = 0.05).	43 patients dropped out/did not complete the follow up at 1 year. Unknown reasons.	1. Antidepressants significantly reduced depression and anxiety score in IBD patients 2. Antidepressants significantly reduced frequency of bowel movements but no other symptoms of IBD.
Walker *et al*. (1996)	2 months	In the 8 patients given the intervention, depression significantly decreased after 8 weeks (*P* < 0.0001)	Not reported	No objective difference in IBD severity	Significant increase in QOL SF-36 score after treatment (*P* < 0.15 to <0.03 for SF-36 subgroups)	Not reported	1. Paroxetine significantly reduced depression score in IBD patients 2. Paroxetine had no effect on IBD severity
Kast (1998)	2 years; follow up at 1 week, 1 month, 2 years + 6 weeks	Depression responded well (no psychometric questionnaires)	Not reported	Complete resolution of abdominal pain; bowels improved from 3 to 4×/day to once per day. Symptoms reemerged after stopping phenelzine	Not reported	Not reported	1. Phenelzine was associated with reduced depressive symptoms in a Crohn’s disease patient 2. Use of phenelzine was associated with improved Crohn’s disease symptom severity
Kast and Altschuler (2001)	Approx. 19 months; regular follow up’s for over 19 months	Major depression remitted, baseline dysthymia remained. At this point, bupropion increased to 150 mg TDS	Not reported	After 19 months: CDAI = 0, abdominal pain eased, 1 well-formed bowel movement daily. Symptoms reemerged after stopping bupropion	Not reported	Not reported	1. Bupropion was associated with remission of depression in a Crohn’s disease patient 2. Bupropion associated with reduction in Crohn’s disease activity
Joshi and Dixit (2013)	6 weeks; follow up at 2 weeks, 6 weeks	Not reported	After 2 weeks, the patient had improvements in anxiety features, and after 6 weeks, relief from anxiety features	After 2 weeks, decreased urgency and reduced tenesmus. After 6 weeks, complete resolution of bloody diarrhoea and rectal pain	Not reported	Not reported	1. Mirtazapine was associated with a reduction in features of anxiety 2. Mirtazapine was associated with a reduction in ulcerative colitis activity

BBTS-I, brief behavioural treatment of sleep in IBD; CDAI, Crohn’s disease activity index; IBD, inflammatory bowel disease; IBDQ, IBD quality of life questionnaire; MCDAI, Mayo clinic disease activity index; SE, standard error; SF-36, short form 36 questionnaire; WHOQOL, WHO quality of life index; WHOQOL-BREF, WHO quality of life index – short version.

### Risk of bias

Risk of bias for the studies is shown in Tables 3 and 4. The CCDAN found that the three RCTs were all at low risk of bias [14–16], as was the nonrandomized trial of bupropion [17]. The nonrandomized trial of tianeptine, however, was at high risk of bias [18]. The NOS showed that both cohort studies were at low risk of bias [19,20].

**Table 3.** CCDAN (Moncrieff *et al*., 2001) score for all included randomized and nonrandomized controlled trials

Study	Daghaghzadeh *et al*. (2015), Iran	Mikocka-Walus *et al*. (2017), Australia	Liang *et al*. (2022), China	Chojnacki *et al*. (2011), Poland	Hashash *et al*. (2022), USA
Objectives and specification of outcomes	2	2	2	2	2
Adequate sample size	0	0	0	1	1
Appropriate duration	1	2	2	2	0
Power calculation	0	2	2	0	2
Method of allocation	2	2	2	1	0
Concealment of allocation	2	2	2	0	0
Clear description of treatments	2	2	2	2	2
Blinding of subjects	1	1	1	1	0
Source of subjects described	2	2	2	0	2
Use of criteria and specification of inclusion criteria	1	1	1	1	1
Exclusion criteria and no. of exclusions	2	2	2	0	2
Description of sample demographics	2	2	1	0	2
Blinding of assessor	1	1	1	0	0
Assessment of compliance	2	0	0	0	1
Details on side-effects	2	2	2	0	0
Record of no. and reasons for withdrawals	2	2	1	0	2
Outcome measures clearly described or validated	2	2	2	2	2
Information on comparability and adjustment	2	1	2	0	0
Inclusion of all subjects in analyses	0	0	0	2	2
Presentation of results with inclusion of data for reanalysis	1	2	2	1	2
Appropriate statistical analysis	2	2	2	2	2
Conclusions justified	1	2	2	1	2
Declaration of interests	2	2	2	0	2
CCDAN total score (high quality >20)	34	36	35	18	29

CCDAN, Cochrane Common Mental Disorders Depression Anxiety and Neurosis group.

**Table 4.** NOS (Wells *et al*., 2022) score for all included cohort studies

Study	Yanartas *et al*. (2016), Turkey	Zanello *et al*. (2020), Italy
Representativeness of the exposed cohort	1	1
Selection of the nonexposed cohort	1	1
Ascertainment of exposure	1	1
Demonstration that outcome of interest was not present at start of study	1	1
Comparability of cohorts on the basis of the design or analysis	1	1
Assessment of outcome	1	1
Was follow-up long enough for outcomes to occur	1	1
Adequacy of follow up of cohorts	1	0
NOS total score (high quality 6–9)	8	7

NOS, Newcastle-Ottawa scale.

### Statistical analysis

#### Primary outcome

**Fig. 2.**
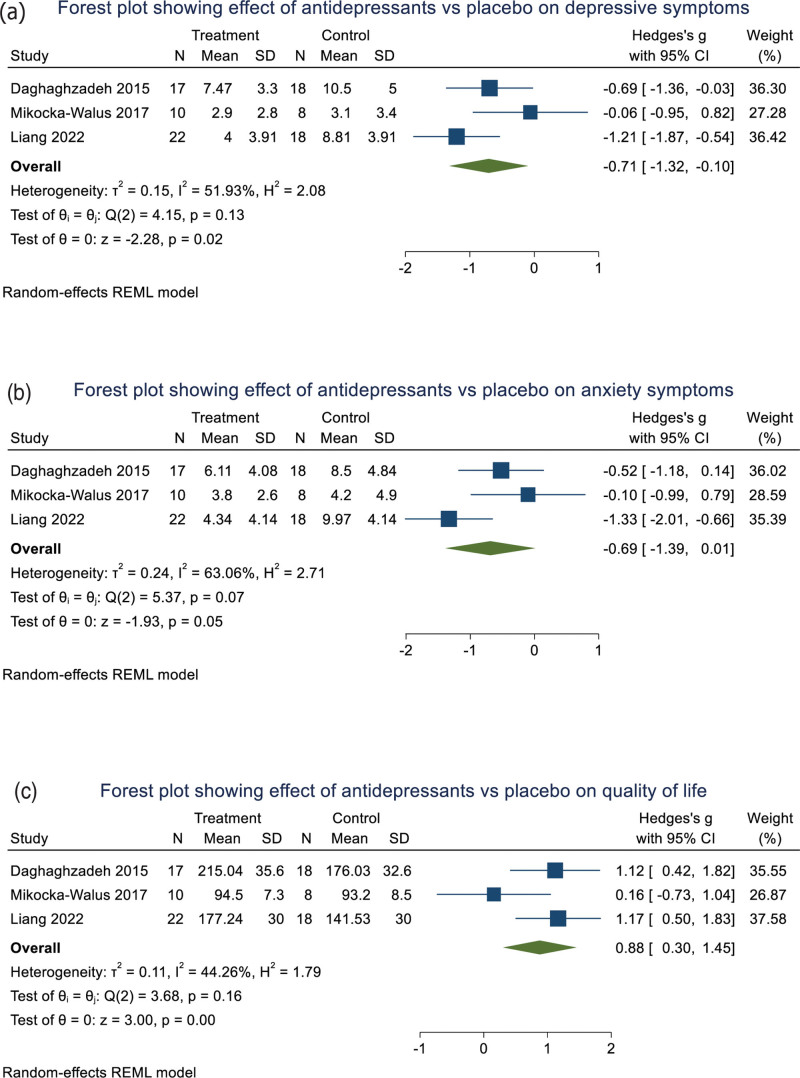
Forest plots showing posttreatment differences between antidepressants and placebo in depressive symptoms (a), anxiety symptoms (b) and quality of life (c).

**Fig. 3.**
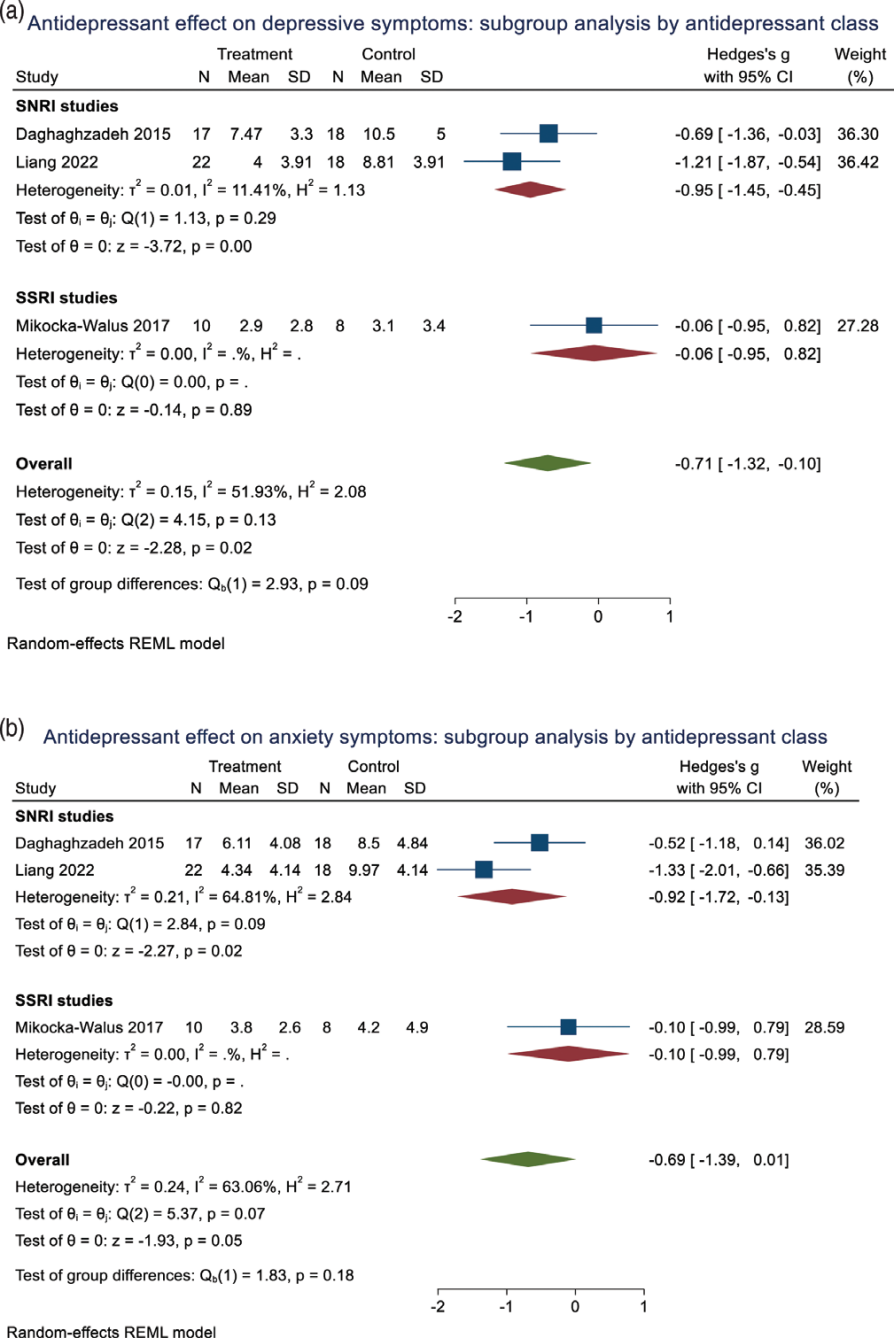
Forest plots showing posttreatment differences between antidepressants and placebo in depression symptoms (a), anxiety symptoms (b) and quality of life (c) stratified by subclass of antidepressant.

Three RCTs were included with 106 participants. In pooled analysis (all using the HADS), posttreatment depressive symptoms were lower in patients taking antidepressants than placebo [SMD = −0.71 (95% confidence interval (CI) −1.32 to −0.10), *P* = 0.02; *I*^2^ = 52%] (Fig. 2a). Analysing by antidepressant class, SNRI antidepressants alone improved depressive symptoms [SMD = −0.95 (95% CI −1.45 to −0.45, *P* < 0.001, *I*^2^ = 11%)] compared to placebo (Fig. 3a).

#### Secondary outcomes

Antidepressants produced a trend improvement in anxiety compared to placebo [SMD = −0.69 (95% CI −1.39 to 0.01), *P* = 0.053; *I*^2^ = 63%] (Fig. 2b). Patients treated with antidepressants exhibited improved QoL compared to those with placebo [SMD = 0.88 (95% CI 0.30–1.45), *P* = 0.003; *I*^2^ = 44%] (Fig. 2c). Analysing by antidepressant class, SNRI antidepressants alone improved anxiety [SMD = −0.92 (95% CI 1.72 to −0.13) (Fig. 3b), *P* = 0.023, *I*^2^ = 65%] and QoL [SMD = 1.14 (95% CI 0.66–1.62), *P* < 0.001, *I*^2^ = 0%] compared to placebo (Fig. 3c).

### Nonrandomized or open-label trials

In a nonrandomized controlled trial, 60 participants with ulcerative colitis in remission were included and allocated equally to tianeptine – an atypical antidepressant – or placebo [18]. After 12 months, those allocated to tianeptine had a significantly improved anxiety symptoms, depressive symptoms and IBD control compared to placebo. In an open-label trial, bupropion – a dopamine and noradrenaline reuptake inhibitor – was not found to improve depressive symptoms or anxiety symptoms [17]. Participants, however, had already completed a psychological intervention prior to starting, leading to a probable floor effect (Table 2).

### Cohort studies

A prospective cohort study found that antidepressant-adherent patients had a significantly greater decrease in anxiety, depressive symptoms and QoL scores compared to nonadherent patients [19]. A small retrospective cohort study found 12-month improvement in depressive symptoms, anxiety symptoms and daily bowel frequency in patients treated with antidepressants, although attrition was high [20] (Table 2).

### Case series and reports

A case series included eight IBD patients with major depressive disorder [21]. Patients were prescribed paroxetine – an SSRI – in an open-label design. At 8 weeks, the patients’ mean depressive symptoms significantly decreased from baseline. Three case reports were included [22–24]. In all three cases, the patients exhibited improved mental health symptoms and IBD disease activity (Table 2).

## Discussion

In this systematic review of antidepressants in IBD, we found 11 studies of 327 participants, including three good-quality RCTs. From the RCTs, we found that antidepressants improved depressive symptoms and QoL scores, whilst producing trend improvement in anxiety symptoms. When analysing SNRI antidepressants alone, significant benefit was seen for depressive symptoms, anxiety symptoms and QoL. Apart from transient side-effects and an increased incidence of nausea in the duloxetine RCT, tolerability was comparable to placebo.

Although antidepressants are effective in the general population, the comorbidity of IBD poses potential barriers to their use. For example, patients with IBD frequently have active gastrointestinal symptoms, chronic pain, elevated systemic inflammation and can have impaired intestinal absorption [5], emphasising the need for clinical trials of antidepressants specifically in IBD. Whilst a recent meta-analysis found benefit of antidepressants for depression in IBD, its findings were primarily based on studies with a high risk of bias [7]. Our findings, therefore, advance the literature by showing that antidepressants are more effective in IBD when meta-analysing based on RCTs.

Symptoms of depression and anxiety are highly prevalent in IBD, affecting approximately 25 and 32% of patients with IBD, respectively [2]. Although these estimates are based on questionnaire scores rather than diagnostic interview, questionnaire-derived depressive symptoms – even at a very low level – are associated with poor IBD outcomes, including increased risk of relapse, hospitalisation and surgery [25]. Whilst depression and anxiety symptoms can result from the psychological distress and burden of IBD, depression frequently predates IBD rather than vice versa [26], suggesting a bidirectional relationship. Furthermore, patients with IBD and comorbid irritable bowel syndrome symptoms – around 25% of those in mucosal remission – have worse symptoms of depression and anxiety [27]. There is, therefore, likely to be a large IBD subgroup with disordered gut–brain interaction, manifesting with psychological symptoms (e.g. depression and anxiety) and gastrointestinal symptoms (e.g. pain and persistent diarrhoea).

Antidepressants have strong potential in IBD, both for their effects on psychological health and for their potential in reducing disordered gut–brain interaction. Use of antidepressants is associated with lower incidence of IBD, suggesting a possible protective effect on the gut-brain axis [26]. SNRIs are effective in improving pain in general [28,29], and it is notable that two of the three successful RCTs used SNRIs, although the low dose of venlafaxine used (150 mg per day) has minimal noradrenergic effects [30]. Only one case report tested mirtazapine. This tetracyclic antidepressant enhances serotonin signalling without increasing serotonin concentrations [31]. It typically has a neutral effect on gut motility, reduces nausea [32], improves insomnia and has a low risk of sexual dysfunction and gastrointestinal bleeds [33]. With only one case report of mirtazapine in IBD to date, our review highlights a need for a trial of mirtazapine in IBD.

Our results also build on a previous systematic review by adding data on adverse events. This is important because side-effects from antidepressants may be more frequently seen in IBD than in the general population [4]. SSRIs present challenges in tolerability to patients with IBD compared to the general population, as they can accelerate gut motility, cause/worsen nausea and increase the risk of gastrointestinal bleeding [32,33]. Conversely, some SSRIs can have anti-inflammatory effects [34]. The SSRI paroxetine, which was used successfully in a small case series in our review, has stronger anticholinergic effects than other SSRIs [35]. By improving diarrhoea, this may improve IBD symptoms more than other SSRIs, but the short half-life of paroxetine increases risk of discontinuation syndrome on cessation [33]. Bupropion, a noradrenaline and dopamine reuptake inhibitor, may be particularly promising in IBD because it marginally slows gut motility, reduces tumor necrosis factor (TNF) concentrations [36], may improve fatigue and is the only antidepressant that improves sexual function [37].

Clinically, our findings support the use of antidepressants in IBD but emphasize more the need for larger, powered clinical trials. Such trials should recruit patients with clinical depression according to diagnostic criteria and include patients in- and out of IBD remission. Trials could target a subgroup of patients, such as those with clinical depression and pain, for whom SNRIs may be particularly beneficial. Bupropion and mirtazapine are particularly promising, based on their pharmacology and clinical effects in other populations. Trials should also be powered to test the moderators and mediators of any improvement in depressive symptoms, such as improvement in IBD symptoms or immune parameters.

Our findings have several limitations. All included studies were modest in size. A number of studies did not specifically select participants with baseline depression or anxiety, despite this being the target population. Trials typically excluded patients with active IBD, which may be a missed opportunity to restore disordered gut–brain interaction.

### Conclusion

Antidepressants show overall benefit for depressive symptoms and quality of life in IBD compared to placebo. SNRI antidepressants, such as duloxetine and venlafaxine, show particular benefit and additional improvement in anxiety. These findings, however, are based on a small number of studies with modest sample sizes. Large, placebo-controlled randomized trials of antidepressants in IBD are now needed.

## Acknowledgements

This report represents independent research part funded by the National Institute for Health Research Biomedical Research Centre and South London and Maudsley National Health Service Foundation Trust and King’s College London. Infrastructure support for this research was provided by the NIHR Imperial Biomedical Research Centre (BRC). The views expressed are those of the authors and not necessarily those of the National Health Service, National Institute for Health Research or Department of Health.

F.W. co-designed the study and wrote the first draft. B.C. performed statistical analyses and revised the draft for important intellectual content. N.P. and A.H.Y. revised the manuscript for important intellectual content. C.D.M. codesigned the study, performed statistical analyses and revised the manuscript for important intellectual content.

All data supporting the authors’ results and conclusions are presented in the manuscript.

### Conflicts of interest

C.D.M. has given paid educational talks for AbbVie, Dr Falk Pharma UK Ltd and Takeda. C.D.M. is funded by an National Institute for Health and Care Research (NIHR) advanced fellowship. N.P. has been speaker, advisory consultant or has received research grants from AbbVie, Allergan, Astra-Zeneca, Bristol-Myers Squibb, Celgene, Celltrion, Dr Falk Pharma UK Ltd, Ferring, Galapagos, GSK, Janssen, Roche, Pfizer, Sobi, Takeda, Tillotts and Vifor. N.P. is funded by the Wellcome Trust. A.H.Y. has served as a consultant, advisory board member or speaker for Flow Neuroscience, Novartis, Roche, Janssen, Takeda, Noema Pharma, Compass, AstraZeneca, Boehringer Ingelheim, Eli Lilly, LivaNova, Lundbeck, Sunovion, Servier, Janssen, Allegan, Bionomics, Sumitomo Dainippon Pharma, Sage and Neurocentrx. For the remaining authors, there are no conflicts of interest.

## Supplementary Material


