# Antidepressant treatment in inflammatory bowel disease: a systematic review and meta-analysis

**DOI:** 10.1097/MEG.0000000000002768

**Published:** 2024-04-29

**Authors:** Frances Weston, Ben Carter, Nick Powell, Allan H. Young, Calum D. Moulton

**Affiliations:** 1 Centre for Affective Disorders, Institute of Psychiatry, Psychology and Neuroscience, King’s College London, London, UK; 2 Department of Biostatistics, Institute of Psychiatry, Psychology and Neuroscience, King’s College London, London, UK; 3 Department of Digestion, Metabolism and Reproduction, Imperial College London, London, UK; 4 South London and Maudsley NHS Foundation Trust, London, UK; 5 Division of Psychiatry, Department of Brain Sciences, Imperial College London; 6 St Mark’s Hospital, London, UK

**Keywords:** Antidepressants, depression, inflammatory bowel disease, systematic review, meta-analysis

## Abstract

**Background:**

Around 25% of patients with inflammatory bowel disease (IBD) have depressive symptoms and 32% have anxiety symptoms. However, the efficacy of antidepressants in IBD has been poorly studied.

**Methods:**

In this systematic review, we searched IBD studies testing antidepressants in four databases (PubMed, Cochrane Library Trials, Embase, Web of Science). The primary outcome was depressive symptoms. Anxiety, IBD disease activity, quality of life (QoL) and adverse events were secondary outcomes. For randomised controlled trials (RCTs), we performed random-effects meta-analysis of the standardised mean difference (SMD) in post-treatment scores between antidepressant and placebo groups. Risk of bias was assessed using the Cochrane Common Mental Disorders Depression Anxiety & Neurosis Group (CCDAN) tool (clinical trials) and Newcastle-Ottawa scale (cohort studies).

**Results:**

Of 4311 studies screened, 11 studies were included (n=327), including three placebo-controlled RCTs, two non-randomised trials, and six other study types. In the pooled analysis of antidepressants (fluoxetine, duloxetine and venlafaxine respectively) versus placebo, antidepressants improved depressive symptoms (SMD=-0.71 [95 CI% -1.32 to -0.10], p=0.02, *I*^2^=51%) and QoL (SMD=0.88 [95% CI 0.30 to 1.45], p=0.003, *I*^2^=44%). Serotonin and noradrenaline reuptake inhibitors (SNRIs) alone improved depressive symptoms (SMD=-0.95 [95% CI -1.45 to -0.45, p<0.001, *I*^2^=11%), anxiety (SMD=-0.92 [95% CI 1.72 to -0.13], p=0.023, *I*^2^=65%) and QoL (SMD=1.14 [95% CI 0.66 to 1.62], p<0.001, *I*^2^=0%). The three RCTs were of good quality.

**Conclusions:**

Based on three small but good-quality studies, antidepressants improve depressive symptoms and QoL compared to placebo in IBD. SNRI antidepressants may also improve anxiety. Owing to the small sample sizes of previous studies, a powered study of antidepressants in IBD is needed.

## Introduction

Inflammatory bowel disease (IBD) is a debilitating, relapsing-remitting illness, which primarily comprises ulcerative colitis (UC) and Crohn’s disease (CD). IBD is associated with unpredictable, painful and often disabling symptoms for patients [1]. Rates of depression are over twice as high in individuals with IBD than in the general population, and these rates are significantly higher for those with active IBD compared to inactive IBD [2]. Notably, the comorbidity of depression is associated with poor IBD outcomes, including increased risk of IBD relapse, hospitalisation, requirement for biologic therapy and surgery [3]. This suggests that effective treatment of depression could improve both quality of life and IBD outcomes.

Antidepressants are an effective treatment for patients with depression and anxiety in the general population and are also commonly used to treat psychological comorbidities in patients with IBD [4]. However, the comorbidity of IBD poses potential barriers to effectiveness and tolerability of antidepressants. These challenges include the burden of IBD symptoms (e.g. pain and diarrhoea), impaired drug absorption [5], and possible increased vulnerability to side-effects in this population [4]. A systematic review of antidepressants in IBD from 2017 was limited by lack of available trials and lack of data on adverse events [6]. A recent meta-analysis found antidepressants to be effective in patients with IBD. However, the findings of this synthesis were based primarily on studies where randomisation or blinding procedures were not reported [7], thereby seriously undermining the validity of the findings. Furthermore, the authors did not analyse by antidepressant class, meaning it remains unclear which specific antidepressants are likely to be most beneficial in patients with IBD.

We therefore performed an updated systematic review and meta-analysis of the effect of antidepressants in patients with IBD, in which we only meta-analysed randomised controlled trials (RCTs). We hypothesised that antidepressants would improve depressive symptoms, anxiety symptoms and quality of life in IBD.

## Methods

The writing of this systematic review was guided by the standards of the Preferred Reporting Items for Systematic Review and Meta-Analysis (PRISMA) Statement [8], in which studies that meet review criteria are examined and RCTs and with sufficient data pooled for meta-analysis.

### Study selection

We included studies that met all the following criteria: 1) recruited participants clinically diagnosed with any type of IBD (CD, UC or indeterminate colitis); 2) were randomised controlled trials, non-randomised controlled trials, case-control studies, prospective and retrospective observational studies, case series or case reports; 3) participants were prescribed any of the following antidepressants at the start of the research: a selective serotonin reuptake inhibitor (SSRI) (sertraline, fluoxetine, paroxetine, fluvoxamine, citalopram, escitalopram), tricyclic antidepressant (amitriptyline, nortriptyline, imipramine, clomipramine, desipramine), tetracyclic antidepressant (mirtazapine, mianserin), serotonin and noradrenaline reuptake inhibitor (SNRI) (venlafaxine, duloxetine), monoamine oxidase inhibitor (moclobemide, phenelzine, selegiline), or other antidepressant (bupropion, vortioxetine, vilazodone, trazodone); and 4) a validated measure of depression was used before and after treatment. Studies were excluded if they met the following criteria: 1) study was qualitative only; 2) study recruited a paediatric population (<18 years); and 3) study was a review article presenting no original data.

### Study extraction

We systematically searched PubMed, Cochrane Library Trials, Embase (via Ovid) and Web of Science for studies published from inception to 31st December 2023. The full search strategy is included as supplementary material. To identify unpublished or ongoing studies, we searched trial registries including ClinicalTrials.gov and the EU clinical trials register. A manual search of references was carried out on the included studies and four reviews in the area of antidepressant use in IBD.

Two authors (F.W. and C.D.M.) independently performed the literature search and resolved differences over inclusion through discussions and consensus. Two reviewers independently screened titles and abstracts for potentially eligible studies and assessed them for inclusion. From abstracts fulfilling inclusion criteria, full-text articles were reviewed, and data extraction performed for studies still meeting inclusion criteria.

### Data extraction

For each study, we extracted the following data: author, year, country, study design, patient age and sex distribution, IBD type, IBD duration, baseline IBD activity (faecal calprotectin, validated disease indices such as the Crohn’s Disease Activity Index [CDAI], serum C-reactive protein), baseline depression and anxiety status, antidepressant treatment (medication, dose, duration of treatment), depression and anxiety after treatment, changes in IBD symptoms or disease activity scores, and frequency of incident adverse effects.

### Risk of bias assessment

Due to the various study designs, two quality assessment methods were used. For RCTs and non-RCTs, we used the Cochrane Common Mental Disorders Depression Anxiety & Neurosis Group (CCDAN) tool, as it has been specifically designed to assess trials related to depressive disorders [9]. The CCDAN consists of 23 items, and gives a score out of 46, with a score of 20 or more suggesting high quality. For cohort studies, the Newcastle-Ottawa scale (NOS) was used, which gives studies a score out of 8, with a higher score signifying lower risk of bias [10].

### Outcomes

The primary outcome was any validated measure of depressive symptoms at the latest time-point within the study. Secondary outcomes were anxiety symptoms, quality of life (QoL), IBD disease activity and adverse events using any validated instrument.

### Measures of effect

For each study, effect size estimates were calculated using the standardized mean difference (SMD) in depressive symptoms after treatment. Where not provided directly, the standard error (SE) of each group sizes was estimated [11].

### Statistical analysis

We considered pooling with a minimum of three RCTs that were clinically homogeneous. Studies were weighted using an inverse-variance method, where studies with larger precision are afforded greater weight. Pooled effect estimates were calculated using a random-effects model [12]. Analyses were performed using STATA 17.0.

### Heterogeneity

Heterogeneity between studies was quantified by calculating the *I*^2^-statistic, *I*^2^ between 25%, 50%, and 75% suggesting low, moderate, and high heterogeneity, respectively [13]. Moderate and high heterogeneity were explored using subgroup analysis by antidepressant class. We did not assess for publication bias due to the small number of studies in the meta-analyses.

## Results

**Figure 1:**
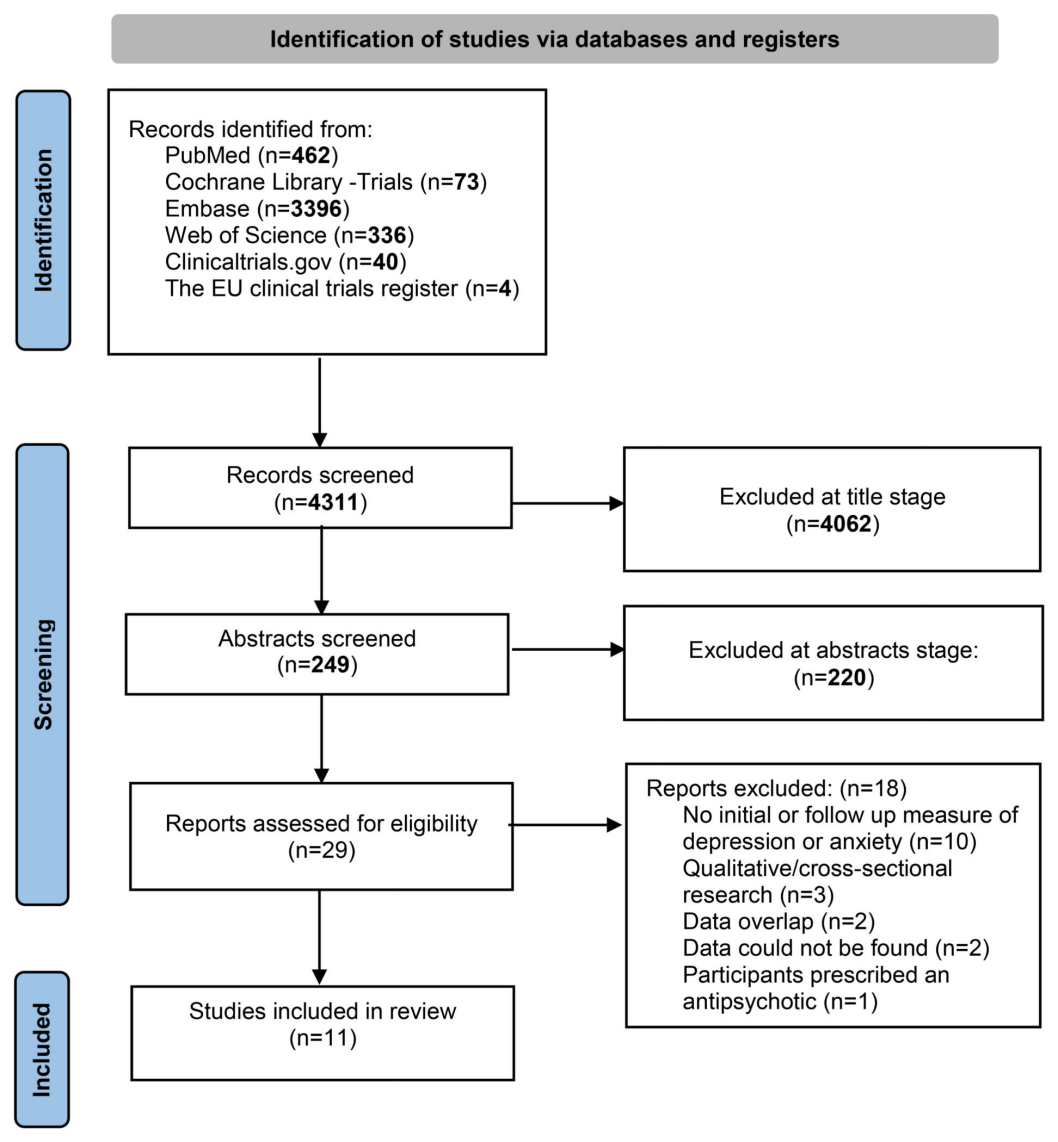
PRISMA flow diagram outlining the systematic review stages

A total of 4311 studies were retrieved and screened for relevance, of which 4282 papers were excluded at title/abstract stage and 29 assessed for eligibility. Following full-text review, 11 studies were included (Figure 1). Table 1 shows the characteristics of included studies.

### Randomized controlled trials

Three small placebo-controlled RCT’s were included. One used the SSRI fluoxetine for 12 months in 26 patients with Crohn’s disease in remission (but with a flare in the last year) [14]; one used the SNRI duloxetine for 6 months in patients with CD or UC in remission [15]; and the other used the SNRI venlafaxine for 6 months in 45 patients with UC or CD, which also included patients with active IBD [16]. For the duloxetine and fluoxetine studies, depression was not an inclusion criterion. For the venlafaxine study, participants required a score ≥8 on the Hospital Anxiety and Depression Scale (HADS), indicative of mild anxiety or depressive symptoms, though did not require a clinical diagnosis of depression.

Following treatment, the duloxetine and venlafaxine trials reported improvement in depression and anxiety scores compared to placebo. There was no significant change in depression or anxiety when using fluoxetine, though baseline depressive symptoms were low and depressive symptoms were a secondary outcome. For IBD control, the venlafaxine study found a significant decrease in both Mayo score and CDAI score after 6 months. The fluoxetine and control CDAI scores and faecal calprotectin levels were comparable at 12 months (Table 2).

### Risk of bias

Risk of bias for the studies is shown in Tables 3 and 4. The CCDAN found that the 3 RCTs were all at low risk of bias [14–16], as was the non-randomised trial of bupropion [17]. However, the non-randomised trial of tianeptine was at high risk of bias [18]. The NOS showed that both cohort studies were at low risk of bias [19,20].

### Statistical analysis

#### Primary outcome

**Figure 2:**
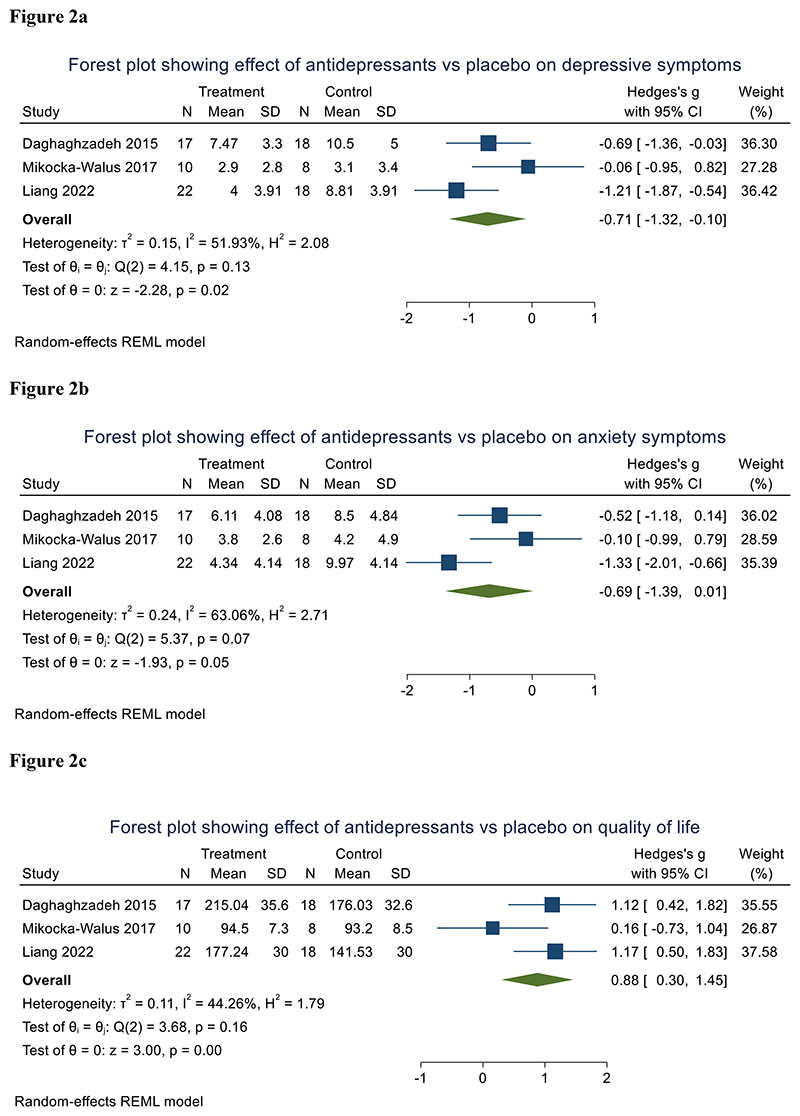
Forest plots showing post-treatment differences between antidepressants and placebo in depressive symptoms (Figure 2a), anxiety symptoms (Figure 2b) and quality of life (Figure 2c).

**Figure 3:**
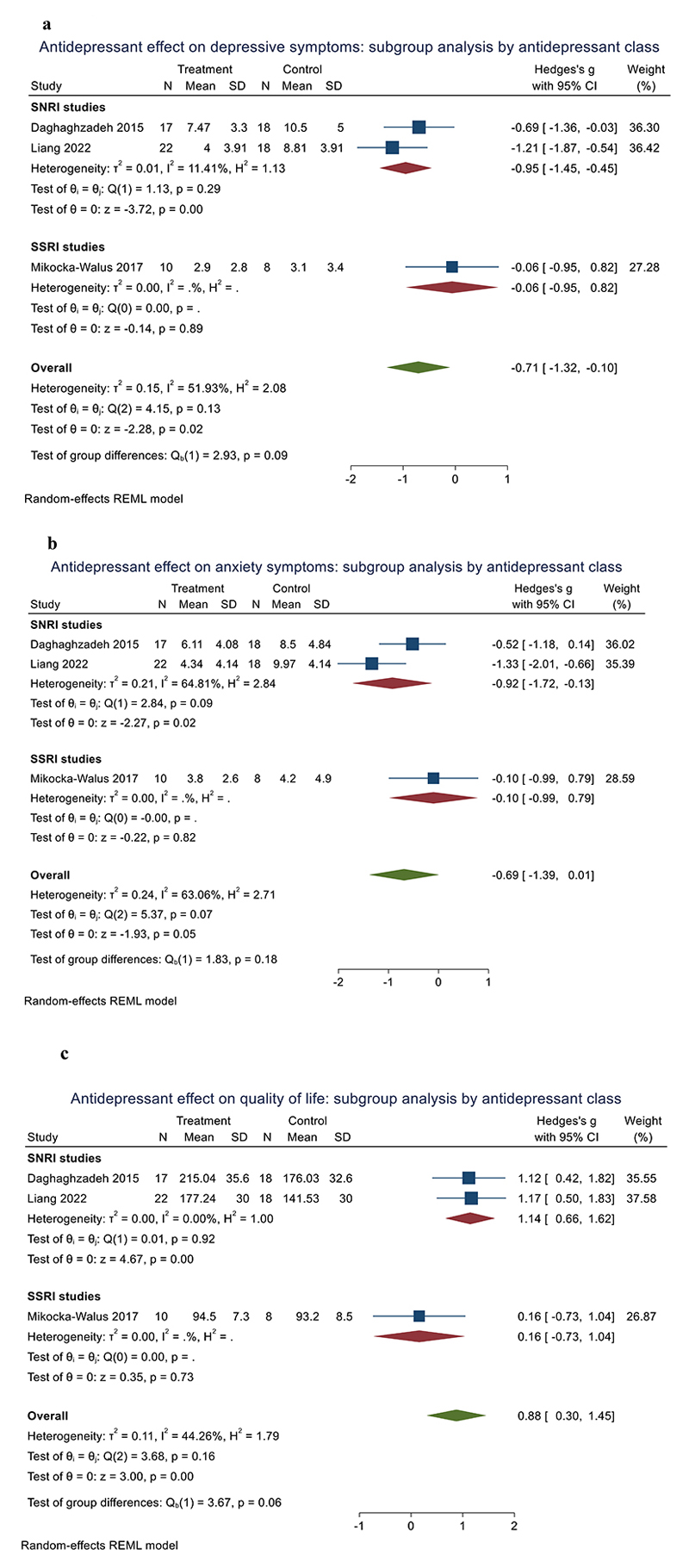
Forest plots showing post-treatment differences between antidepressants and placebo in depression symptoms (Figure 3a), anxiety symptoms (Figure 3b) and quality of life (Figure 3c), stratified by sub-class of antidepressant.

Three RCTs were included with 106 participants. In pooled analysis (all using the Hospital Anxiety and Depression Scale [HADS]), post-treatment depressive symptoms were lower in patients taking antidepressants than placebo (SMD=-0.71 [95% CI - 1.32 to -0.10], p=0.02; *I*^2^=51%) (Figure 2a). Analysing by antidepressant class, SNRI antidepressants alone improved depressive symptoms (SMD=-0.95 [95% CI -1.45 to -0.45, p<0.001, *I*^2^=11%) compared to placebo (Figure 3a).

#### Secondary outcomes

Antidepressants produced a trend improvement in anxiety compared to placebo (SMD=-0.69 [95% CI -1.39 to 0.01], p=0.053; *I*^2^=63%) (Figure 2b). Patients treated with antidepressants exhibited improved QoL compared to those with placebo (SMD=0.88 [95% CI 0.30 to 1.45], p=0.003; *I*^2^=44%) (Figure 2c). Analysing by antidepressant class, SNRI antidepressants alone improved anxiety (SMD=-0.92 [95% CI 1.72 to -0.13] (Figure 3b), p=0.023, *I*^2^=65%) and QoL (SMD=1.14 [95% CI 0.66 to 1.62], p<0.001, *I*^2^=0%) compared to placebo (Figure 3c).

### Non-randomized or open-label trials

In a non-randomized controlled trial, 60 participants with UC in remission were included and allocated equally to tianeptine – an atypical antidepressant – or placebo [18]. After 12 months, those allocated to tianeptine had a significantly improved anxiety symptoms, depressive symptoms and IBD control compared to placebo. In an open-label trial, bupropion – a dopamine and noradrenaline reuptake inhibitor – was not found to improve depressive symptoms or anxiety symptoms [17]. However, participants had already completed a psychological intervention prior to starting, leading to a probable floor effect (Table 2).

### Cohort Studies

A prospective cohort study found that antidepressant-adherent patients had a significantly greater decrease in anxiety, depressive symptoms and QoL scores compared to non-adherent patients [19]. A small retrospective cohort study found 12-month improvement in depressive symptoms, anxiety symptoms and daily bowel frequency in patients treated with antidepressants, although attrition was high [20] (Table 2).

### Case series and reports

A case series included 8 IBD patients with major depressive disorder [21]. Patients were prescribed paroxetine – an SSRI – in an open-label design. At 8 weeks, the patients’ mean depressive symptoms significantly decreased from baseline. Three case reports were included [22–24]. In all three cases, the patients exhibited improved mental health symptoms and IBD disease activity (Table 2).

## Discussion

In this systematic review of antidepressants in IBD, we found 11 studies of 327 participants, including three good-quality RCTs. From the RCTs, we found that antidepressants improved depressive symptoms and QoL scores, whilst producing trend improvement in anxiety symptoms. When analysing SNRI antidepressants alone, significant benefit was seen for depressive symptoms, anxiety symptoms and QoL. Apart from transient side-effects and an increased incidence of nausea in the duloxetine RCT, tolerability was comparable to placebo.

Although antidepressants are effective in the general population, the comorbidity of IBD poses potential barriers to their use. For example, patients with IBD frequently have active gastrointestinal symptoms, chronic pain, elevated systemic inflammation and can have impaired intestinal absorption [5], emphasising the need for clinical trials of antidepressants specifically in IBD. Whilst a recent meta-analysis found benefit of antidepressants for depression in IBD, its findings were primarily based on studies with a high risk of bias [7]. Our findings therefore advance the literature by showing that antidepressants are more effective in IBD when meta-analysing based on RCTs.

Symptoms of depression and anxiety are highly prevalent in IBD, affecting approximately 25% and 32% of patients with IBD respectively [2]. Although these estimates are based on questionnaire scores rather than diagnostic interview, questionnaire-derived depressive symptoms – even at a very low level – are associated with poor IBD outcomes, including increased risk of relapse, hospitalisation and surgery [25]. Whilst depression and anxiety symptoms can result from the psychological distress and burden of IBD, depression frequently predates IBD rather than vice versa [26], suggesting a bidirectional relationship. Furthermore, patients with IBD and comorbid irritable bowel syndrome symptoms – around 25% of those in mucosal remission – have worse symptoms of depression and anxiety [27]. There is therefore likely to be a large IBD subgroup with disordered gut-brain interaction, manifesting with psychological symptoms (e.g. depression and anxiety) and GI symptoms (e.g. pain and persistent diarrhoea).

Antidepressants have strong potential in IBD, both for their effects on psychological health and for their potential in reducing disordered gut-brain interaction. Use of antidepressants is associated with lower incidence of IBD, suggesting a possible protective effect on the gut-brain-axis [26]. SNRIs are effective in improving pain in general [28,29], and it is notable that two of the three successful RCTs used SNRIs, although the low dose of venlafaxine used (150mg per day) has minimal noradrenergic effects [30]. Only one case report tested mirtazapine. This tetracyclic antidepressant enhances serotonin signalling without increasing serotonin concentrations [31]. It typically has a neutral effect on gut motility, reduces nausea [32], improves insomnia and has a low risk of sexual dysfunction and gastrointestinal bleeds [33]. With only one case report of mirtazapine in IBD to date, our review highlights a need for a trial of mirtazapine in IBD.

Our results also build on a previous systematic review by adding data on adverse events. This is important because side-effects from antidepressants may be more frequently seen in IBD than in the general population [4]. SSRIs present challenges in tolerability to patients with IBD compared to the general population, as they can accelerate gut motility, cause/worsen nausea and increase the risk of gastrointestinal bleeding [32,33]. Conversely, some SSRIs can have anti-inflammatory effects [34]. The SSRI paroxetine, which was used successfully in a small case series in our review, has stronger anticholinergic effects than other SSRIs [35]. By improving diarrhoea, this may improve IBD symptoms more than other SSRIs, but the short half-life of paroxetine increases risk of discontinuation syndrome on cessation [33]. Bupropion, a noradrenaline and dopamine reuptake inhibitor, may be particularly promising in IBD because it marginally slows gut motility, reduces TNF concentrations [36], may improve fatigue and is the only antidepressant that improves sexual function [37].

Clinically, our findings support the use of antidepressants in IBD but emphasise more the need for larger, powered clinical trials. Such trials should recruit patients with clinical depression according to diagnostic criteria and include patients in- and out of IBD remission. Trials could target a subgroup of patients, such as those with clinical depression and pain, for whom SNRIs may be particularly beneficial. Bupropion and mirtazapine are particularly promising, based on their pharmacology and clinical effects in other populations. Trials should also be powered to test the moderators and mediators of any improvement in depressive symptoms, such as improvement in IBD symptoms or immune parameters.

Our findings have several limitations. All included studies were modest in size. A number of studies did not specifically select participants with baseline depression or anxiety, despite this being the target population. Trials typically excluded patients with active IBD, which may be a missed opportunity to restore disordered gut-brain interaction.

## Conclusion

Antidepressants show overall benefit for depressive symptoms and quality of life in IBD compared to placebo. SNRI antidepressants, such as duloxetine and venlafaxine, show particular benefit and additional improvement in anxiety. However, these findings are based on a small number of studies with modest sample sizes. Large, placebo-controlled randomised trials of antidepressants in IBD are now needed.

## Supplementary Material

Supplementary material

## Figures and Tables

**Table 1:** Characteristics of included studies

Author (year), country	Study design	Sample size, sex, mean age (SD)	IBD type; mean duration (SD)	IBD baseline measures & mean score (SD)	IBD medication	Baseline depression & anxiety measure: mean score (SD)	Antidepressant type, dose, & duration
Daghaghzadeh et al. (2015), Iran	Placebo-controlled RCT	N=35: 19 female, 16 male. 38 years (8.2)	UC: 22, CD: 13; 6.49 years (3.27)	LCAI for both UC and CD 6.88 (3.8) with no flair in prior 6 months	Both groups given mesalazine 2-4 g/day	HADS-D 9.22 (3.45). HADS-A: 8.17 (4.29).	Intervention: duloxetine 30mg/day for 1 week, then 60 mg/day for next 11 weeks. Placebo: same form and packaging as intervention.
Mikocka-Walus et al. (2017), Australia	Placebo-controlled RCT	N=26; 12 female, 14 male. 37.4 years (13.2)	All CD intervention: 14.98 years (13.1) placebo: 12.21 years (8.1)	CDAI intervention: 63.8 (44.4) placebo: 66.4 (44.7) Faecal calprotectin intervention: 46.4 (33.2) placebo: 98.1 (94.4) All in remission but flare in last 12 months.	Remained on current CD medication: complementary (n=12), mesalazine (n=7), biologics (n=13), immunomodulators (n=19), prednisolone (n=1). Medication unchanged throughout.	HADS-D: intervention: 3.9 (2.9), placebo: 3.6 (3.1). HADS-A: intervention: 5.3 (4.1), placebo: 4.9 (3.4)	Intervention: fluoxetine 20mg/day. Placebo: gelatin capsules filled with microcrystalline cellulose
Liang et al. (2022), China	Placebo-controlled RCT	N=45: 20 female, 25 male. 40 years (13.12)	UC: 22 years CD: 23 years	Mayo score for UC: 5.80 (3.81). CDAI for CD: 268.02 (119.8).	Remained on current IBD medication: mesalazine (n=14), corticosteroids (n=3), immunomodulators (n=12), biologics (n=16)	HADS (MDD): intervention: 8.81, placebo: 8.81 HADS (GAD): intervention: 10.09, placebo: 10.09	Intervention: venlafaxine (sustained-release form) 75mg/day for 1 week, then increased to 150 mg. Placebo: gelatin capsules filled with starch
Chojnacki et al. (2011), Poland	Non-randomised controlled trial	N=60: 39 female, 21 male. 30.6 years (8.8)	All UC Range 4-16 years	In remission for prior 6 months, confirmed endoscopically and using Mayo index	Both groups given 2.0 g of aminosalicylates/ day	BDI: 19.95 (4.49) HAMA: 20.35 (4.03)	Intervention: 2.0g aminosalicylates and tianeptine 37.5mg/day for 12 months. Placebo: 2.0 g aminosalicylates and a placebo for 12 months
Hashash et al. (2022), USA	Open-label trial	N=68: 41 female, 27 male. 23.8 years (4.8)	All CD (duration unknown)	HBI mild or no disease activity: 4.30 (5.0). At 4 weeks, Bupropion + BBTS-I: 4.7 (5.2), BBTS-I Only: 3.7 (4.9)	Remained on current CD medications: aminosalicylates (n=21), biologics (n=35), steroids (n=9), immunomodulators (n=34)	HDRS – baseline: 12.62 (5.6). At 4 weeks -bupropion + BBTS-I: 12.7 (5.6), BBTS-I only: 10.7 (4.8). HARS at baseline: 13.86 (7.9). At 4 weeks, bupropion + BBTS-I: 14.6 (8.0), BBTS-I only: 9.6 (5.3)	Intervention: bupropion sustained release 100–300mg/day for 8 weeks in patients continuing to experience fatigue after behavioural therapy alone (n=33).
Yanartas et al. (2016), Turkey	Prospective cohort	N=67: 43 female, 24 male. 40.71 years (12.71)	UC: 36, CD: 31	CDAI group A: 197.41 (130.60), group B: 58.50 (74.94). MMS: group A: 2.71 (3.05), group B: 2.78 (3.42).	Remained on current IBD medication: mesalamine (n=45), corticosteroids (n=4), anti-TNF (n=25), azathioprine (n=29), methotrexate (n=8), budesonide (n=3)	HADS (MDD) 43.3%. Group A: 10.62 (3.61). Group B: 11.55 (2.85). HADS (GAD) 15%. Group A: 12.38 (4.38). Group B: 11.40 (4.60).	Sertraline 21.0%, escitalopram 15.8%, bupropion 12.3%, mirtazapine 12.3%, paroxetine 10.6%, venlafaxine 5.2%, duloxetine 3.5%, fluvoxamine 1.8% (sertraline + mirtazapine) 7.0%, (escitalopram + trazodone) 5.2%, (venlafaxine + mirtazapine) 1.8%), and (sertraline + quetiapine) 3.5%.
Zanello et al. (2020), Italy	Retrospective cohort	N=15: 9 females, 6 male. 48.27 years (13.7)	UC: 6, CD: 9 31.3 years (13.3)	CDAI or HBI for CD. Partial Mayo score for UC. In clinical remission.	Remained on current IBD medication: NSAIDs (n=5), immunosuppressive (n=1), TNF inhibitors (n=5), polytherapy (n=3)	HAM-D: 21.5 (7.4). HAM-A: 25.6 (8.1)	Sertraline (n=10), citalopram (n=1), paroxetine (n=1), fluoxetine (n=1), duloxetine (n=2)
Walker et al. (1996), USA	Case Series	N=8 43.1 years (11.3)	IBD – type unknown	Structured gastrointestinal symptoms interview	Not reported	HAM-D: 29.0 (7.8). Anxiety: N/A.	Paroxetine 20-40mg OD according to response
Kast (1998), USA	Case report	N=1 female, 33 years	CD, duration unknown	10 watery bowel movements with severe abdominal cramping daily, dietary intolerance to all solid foods	5 mg azathioprine, 60 mg prednisone, and 3 acetaminophen/oxycodone tablets daily. At 1 month, azathioprine and prednisone tapered off.	Anxiety-prominent major depressive episode	Phenelzine 45-90mg/day according to response
Kast & Altschuler (2001), USA	Case report	N=1 female, 44 years	CD, duration unknown	CDAI: 202. Pain throughout week. Bleeding and incontinence.	500 mg mesalamine 2x/day, tapered off after a few months	Major depression, superimposed on a chronic mild depressed state (dysthymia).	Fluoxetine 40 mg/day for depression changed to bupropion 150mg twice a day, increased to 150mg 3 times a day
Joshi & Dixit (2013), India	Case report	N=1 male, 26 years	UC, duration unknown	Urgency of defecation, bloody diarrhoea and rectal pain. Past courses of steroids had minimal benefit.	Immunomodulators, and steroids in past. Past courses of steroids had minimal benefit.	Features of GAD	Mirtazapine 15 mg at night

BDI: Beck Depression Inventory, CD: Crohn's disease, CDAI: Crohn’s Disease Activity Index, GAD: generalised anxiety disorder, HADS: The Hospital Anxiety and Depression Scale, HAD-A: The Hospital Anxiety and Depression Scale - Anxiety HAD-D: The Hospital Anxiety and Depression Scale – Depression, HBI: Harvey-Bradshaw index, HAMA: The Hamilton Anxiety Scale, HARS: Hamilton Anxiety Rating Scale, HAM-A: Hamilton Rating Scale for Anxiety, HAM-D: Hamilton Rating Scale for Depression, HDRS: Hamilton Depression Rating Scale, IBD: Inflammatory Bowel Disease, LCAI: Lichtiger Colitis Activity Index, MCDAI: Mayo Clinic Disease Activity Index, MMS: Modified mayo score, MDD: Major Depressive Disorder, RCT: randomized controlled trial, UC: Ulcerative Colitis.

**Table 2:** Summary of study results and outcomes for IBD papers

Author (year)	Follow-up duration	Changes in depression after treatment	Changes in anxiety and fatigue	Changes in IBD symptoms or control	Changes in quality of life	Significant adverse effects	Outcomes
Daghaghzadeh et al. (2015)	3 months	Greater decrease in depression with intervention vs placebo (p=0.041)	Greater decrease in anxiety with intervention vs placebo (p=0.049)	Greater decrease in severity of symptoms with intervention vs placebo (p=0.02)	Greater increase in psychological WHOQOL score with intervention vs placebo (p=0.038), using the WHOQOL-BREF. Sub-domain changes: physical (p=0.001), social (p=0.015), environmental (p=0.26)	n=1 stopped duloxetine due to SE’s. Nausea more frequent in duloxetine (n=7) vs placebo (n=2) (p=0.049). Other side effects showed not different between duloxetine and placebo: dizziness (n=4, n=1), drowsiness (n=3, n=0), sexual side effects (n=2, n=0), insomnia (n=2, n=0)	Duloxetine significantly reduced depression and anxiety score in IBD patients. Duloxetine also significantly decreased IBD symptom severity
Mikocka-Walus et al. (2017)	12 months; follow up at 3, 6, & 12 months	No significant group difference in depression score over 12 months (p=0.96)	No significant group difference in anxiety score over 12 months (p=0.98)	No significant difference between fluoxetine and control CDAI score (p=0.98) or faecal calprotectin (p=0.37) over 12 months. No difference in proportion in remission at any time point (p<0.05)	No significant group difference in psychological WHOQOL score (p=0.88). Sub-domain changes: physical QOL (p=0.65), social relationships (p=0.65), and environmental (p=0.98)	N=1 stopped due to side-effects of intervention. All side-effects resolved within first 2 weeks. Intervention SE’s: fatigue (n=4), low mood/anxiety (n=2); nausea, diarrhoea or vomiting (n=2), dry mouth (n=1), hot flushes (n=1). Placebo AEs: muscle spasm (n=2), nausea, diarrhoea or vomiting (n=1).	Fluoxetine had no significant effect on depression or anxiety in CD. Fluoxetine had no significant effect on CD activity.
Liang et al. (2022)	6 months; follow up at 3 & 6 months	Greater decrease in depression with intervention vs placebo at 3 months (p< 0.001) and 6 months (p <0.001)	Greater decrease in anxiety with intervention vs to placebo at 3 months (p<0.001) and 6 months (p<0.001)	UC group in intervention improved in Mayo score vs placebo at 6 months (p <0.001). CD group improved in CDAI vs placebo at 6 months (p=0.006)	Greater increase in IBDQ score in intervention vs placebo (p<0.001)	Intervention: dizziness (n=3), palpitation (n=1), nausea (n=2), and insomnia (n=1). Placebo: nausea (n=1) and diarrhoea (n=1)	Venlafaxine significantly reduced depression and anxiety score in IBD patients Venlafaxine also significantly reduced IBD disease activity after 6 months
Chojnacki et al. (2011)	12 months; follow up at 3, 6, 9 & 12 months	Greater decrease in depression with tianeptine vs placebo at 6 (p<0.05), 9 (p<0.01) & 12 months (p<0.001)	Greater decrease in anxiety with tianeptine vs placebo after 9 (p<0.001) and 12 months of treatment (p<0.001)	MCDAI improved for tianeptine vs placebo (p<0.01). No change in CRP for placebo between groups	Not reported	Intervention: nausea in week 1 (n=4), & mild headaches (n=3), none required discontinuation. Placebo: episodic headaches (n=4)	Tianeptine significantly reduced depression and anxiety score in UC patients Tianeptine also significantly decreased UC disease activity
Hashash et al. (2022)	3 months: latter 2 months with intervention	Significant decrease in depression in intervention group (p=0.001). In the BBTS-I only group, significant decrease in depression (p=0.02)	Significant decrease in anxiety following intervention (p=0.008). In the BBTS-I only group, a significant decrease in anxiety (p=0.02)	No significant change in HBI score in Bupropion + BBTS-I (p=0.65) or BBTS-I only group (p=0.39) over 8 weeks	Not reported	4 subjects in the bupropion group never took or stopped medication within 30 days because they felt that it was not needed (n = 2), made them feel anxious (n = 1) or felt no benefit (n = 1).	Bupropion significantly reduced depression and anxiety score in CD patients Bupropion had no significant effect on CD activity.
Yanartas et al. (2016)	6 months	Greater improvement in depression in group adherent to antidepressant treatment vs patients nonadherent (p=0.017)	Greater improvement in depression in group adherent to antidepressant treatment vs patients nonadherent (p=0.001)	Significant decrease in mean CDAI score (SD) for antidepressant-adherent patients (p=0.011) but no between-group differences	Greater increase in QOL SF-36 in group adherent to anti-depressant treatment vs patients nonadherent to anti-depressants (p=0.010 to <0.001 for each QOL SF-36 subgroup)	1/3 experienced side-effects: drowsiness and fatigue (n=5), sexual dysfunction (n=4), weight gain (n=4), insomnia (n=3), anxiety (n=2), nausea (n=1).	Antidepressants significantly reduced depression and anxiety score in IBD patients Antidepressants significantly reduced IBD activity
Zanello et al. (2020)	12 months; follow up at 1, 3, 6 & 12 months	Depression significantly decreased over 12 months (p=0.001)	Anxiety significantly decreased over 12 months (p=0.001)	Frequency of bowel movements significantly decreased over 12 months (p=0.003). No significant change in abdominal pain or bleeding	Greater increase in QOL SF-36 in those with an anxiety or mood disorder in the “Vitality” domain (p=0.03), “General Health Perception” (p=0.05) and “Mental Health” (p=0.05).	43 patients dropped out/did not complete the follow up at 1 year. Unknown reasons.	Antidepressants significantly reduced depression and anxiety score in IBD patients Antidepressants significantly reduced frequency of bowel movements but no other symptoms of IBD.
Walker et al. (1996)	2 months	In the 8 patients given the intervention, depression significantly decreased after 8 weeks (p<0.0001)	Not reported	No objective difference in IBD severity	Significant increase in QOL SF-36 score after treatment (p<0.15 to <0.03 for SF-36 subgroups)	Not reported	Paroxetine significantly reduced depression score in IBD patients Paroxetine had no effect on IBD severity
Kast (1998)	2 years; follow up at 1 week, 1 month, 2 years + 6 weeks	Depression responded well (no psychometric questionnaires)	Not reported	Complete resolution of abdominal pain; bowels improved from 3-4x/day to once per day. Symptoms re-emerged after stopping phenelzine	Not reported	Not reported	Phenelzine was associated with reduced depressive symptoms in a CD patient Use of phenelzine was associated with improved CD symptom severity
Kast & Altschuler (2001)	Approx. 19 months; regular follow up’s for over 19 months.	Major depression remitted, baseline dysthymia remained. At this point, bupropion increased to 150mg TDS	Not reported	After 19 months: CDAI=0, abdominal pain eased, 1 well-formed bowel movement daily. Symptoms re-emerged after stopping bupropion	Not reported	Not reported	Bupropion was associated with remission of depression in a CD patient Bupropion associated with reduction in CD activity
Joshi & Dixit (2013)	6 weeks; follow up at 2 weeks, 6 weeks	Not reported	After 2 weeks, the patient had improvements in anxiety features, and after 6 weeks, relief from anxiety features.	After 2 weeks, decreased urgency and reduced tenesmus. After 6 weeks, complete resolution of bloody diarrhoea and rectal pain.	Not reported	Not reported	Mirtazapine was associated with a reduction in features of anxiety Mirtazapine was associated with a reduction in UC activity

BBTS-I: Brief Behavioural Treatment of Sleep in IBD, CD: Crohn's disease, CDAI: Crohn’s Disease Activity Index, Harvey-Bradshaw index, HAMA: The Hamilton Anxiety Scale, IBD: Inflammatory Bowel Disease, IBDQ: IBD Quality of Life Questionnaire, LCAI: Lichtiger Colitis Activity Index, MCDAI: Mayo Clinic Disease Activity Index, MMS: Modified mayo score, MDD: Major Depressive Disorder, RCT: randomized controlled trial, SF-36: Short Form 36 Questionnaire, UC: Ulcerative Colitis, WHOQOL: World Health Organisation Quality of Life Index, WHOQOL-BREF: World Health Organisation Quality of Life Index – Short Version

**Table 3:** Cochrane Common Mental Disorders Depression Anxiety & Neurosis Group (CCDAN) (Moncrieff et al., 2001) score for all included randomized and non-randomized controlled trials

Study:	Daghaghzadeh et al. (2015), Iran	Mikocka-Walus et al. (2017), Australia	Liang et al. (2022), China	Chojnacki et al. (2011), Poland	Hashash et al. (2022), USA
					
1. Objectives & specification of outcomes	2	2	2	2	2
2. Adequate sample size	0	0	0	1	1
3. Appropriate duration	1	2	2	2	0
4. Power calculation	0	2	2	0	2
5. Method of allocation	2	2	2	1	0
6. Concealment of allocation	2	2	2	0	0
7. Clear description of treatments	2	2	2	2	2
8. Blinding of subjects	1	1	1	1	0
9. Source of subjects described	2	2	2	0	2
10. Use of criteria & specification of inclusion criteria	1	1	1	1	1
11. Exclusion criteria & no. of exclusions	2	2	2	0	2
12. Description of sample demographics	2	2	1	0	2
13. Blinding of assessor	1	1	1	0	0
14. Assessment of compliance	2	0	0	0	1
15. Details on side-effects	2	2	2	0	0
16. Record of no. & reasons for withdrawals	2	2	1	0	2
17. Outcome measures clearly described or validated	2	2	2	2	2
18. Information on comparability & adjustment	2	1	2	0	0
19. Inclusion of all subjects in analyses	0	0	0	2	2
20. Presentation of results with inclusion of data for reanalysis	1	2	2	1	2
21. Appropriate statistical analysis	2	2	2	2	2
22. Conclusions justified	1	2	2	1	2
23. Declaration of interests	2	2	2	0	2
**CCDAN TOTAL SCORE (high quality >20)**	**34**	**36**	**35**	**18**	**29**

**Table 4:** Newcastle Ottawa Scale (NOS) (Wells et al., 2022) score for all included cohort studies

Study:	Yanartas et al. (2016), Turkey	Zanello et al. (2020), Italy
		
1. Representativeness of the exposed cohort	1	1
2. Selection of the non-exposed cohort	1	1
3. Ascertainment of exposure	1	1
4. Demonstration that outcome of interest was not present at start of study	1	1
5. Comparability of cohorts on the basis of the design or analysis	1	1
6. Assessment of outcome	1	1
7. Was follow-up long enough for outcomes to occur	1	1
8. Adequacy of follow up of cohorts	1	0
**NOS TOTAL SCORE (high quality 6-9)**	**8**	**7**

## Data Availability

All data supporting the authors’ results and conclusions are presented in the manuscript.
