# Supplementary material for: Antidepressant treatment in inflammatory bowel disease: a systematic review and meta-analysis
Source: Eur J Gastroenterol Hepatol. 2024 Apr 29;36(7):850–60. doi: 10.1097/MEG.0000000000002768 (PMC11136269; doi:10.1097/MEG.0000000000002768)
Supplement: Supplementary file 1 [file ejgh-36-850-s001.pdf]

Supplementary material

Search strategy:

PubMed

Search
(antidepressant OR anti-depressant OR antidepressants OR ssri OR sertraline OR fluoxetine OR paroxetine OR fluvoxamine OR citalopram OR escitalopram OR tricyclic OR tricyclics OR amitriptyline OR nortriptyline OR imipramine OR clomipramine OR desipramine OR mirtazapine OR mianserin OR snri OR venlafaxine OR duloxetine OR bupropion OR vortioxetine OR maoi OR moclobemide OR phenelzine OR selegiline OR vilazodone OR trazodone) AND (crohn's OR crohns OR colitis OR IBD OR "inflammatory bowel" OR "short bowel" OR "intestinal failure" OR "short gut" OR "parenteral nutrition") NOT (rat OR rats OR mouse OR mice)

Cochrane Library - Trials

Search
(antidepressant OR anti-depressant OR antidepressants OR ssri OR sertraline OR fluoxetine OR paroxetine OR fluvoxamine OR citalopram OR escitalopram OR tricyclic OR tricyclics OR amitriptyline OR nortriptyline OR imipramine OR clomipramine OR desipramine OR mirtazapine OR mianserin OR snri OR venlafaxine OR duloxetine OR bupropion OR vortioxetine OR maoi OR moclobemide OR phenelzine OR selegiline OR vilazodone OR trazodone) AND (crohn's OR crohns OR colitis OR IBD OR "inflammatory bowel" OR "short bowel" OR "intestinal failure" OR "short gut" OR "parenteral nutrition")

Embase

#	**Searches**
1	antidepressant.mp. or exp antidepressant agent/ or anti-depressant.mp.
2	ssri.mp. or exp serotonin reuptake inhibitor/
3	(sertraline or fluoxetine or paroxetine or fluvoxamine or citalopram or escitalopram or amitriptyline or nortriptyline or imipramine or clomipramine or desipramine or mirtazapine or mianserin or venlafaxine or duloxetine or bupropion or vortioxetine or moclobemide or phenelzine or selegiline or vilazodone or trazodone).mp. [mp=title, abstract, heading word, drug trade name, original title, device manufacturer, drug manufacturer, device trade name, keyword heading word, floating subheading word, candidate term word]
4	snri.mp. or exp serotonin noradrenalin reuptake inhibitor/

5	(tricyclic or tricyclics).mp. [mp=title, abstract, heading word, drug trade name, original title, device manufacturer, drug manufacturer, device trade name, keyword heading word, floating subheading word, candidate term word]
6	exp monoamine oxidase inhibitor/ or maoi.mp.
7	exp inflammatory bowel disease/ or IBD.mp.
8	crohn*.mp.
9	exp colitis/
10	exp short bowel syndrome/ or short bowel.mp.
11	exp intestinal failure/
12	short gut.mp.
13	parenteral nutrition.mp. or exp parenteral nutrition/
14	1 or 2 or 3 or 4 or 5 or 6
15	7 or 8 or 9 or 10 or 11 or 12 or 13
16	14 and 15
17	limit 16 to human

Web of science

#	Search
1	((((((((((((((((((((((((((ALL=(antidepressant)) OR ALL=(anti-depressant)) OR ALL=(antidepressants)) OR ALL=(ssri)) OR ALL=(sertraline)) OR ALL=(fluoxetine)) OR ALL=(paroxetine)) OR ALL=(fluvoxamine)) OR ALL=(citalopram)) OR ALL=(escitalopram)) OR ALL=(tricyclic)) OR ALL=(tricyclics)) OR ALL=(amitriptyline)) OR ALL=(nortriptyline)) OR ALL=(imipramine)) OR ALL=(clomipramine)) OR ALL=(desipramine)) OR ALL=(mirtazapine)) OR ALL=(mianserin)) OR ALL=(snri)) OR ALL=(venlafaxine)) OR ALL=(duloxetine)) OR ALL=(bupropion)) OR ALL=(vortioxetine)) OR ALL=(maoi)) OR ALL=(moclobemide)) OR ALL=(phenelzine)) OR ALL=(selegiline)) OR ALL=(vilazodone)) OR ALL=(trazodone)
2	((((((((ALL=(crohn’s)) OR ALL=(crohns)) OR ALL=(colitis)) OR ALL=(IBD)) OR ALL=("inflammatory bowel")) OR ALL=("short bowel")) OR ALL=("intestinal failure")) OR ALL=("short gut")) OR ALL=("parenteral nutrition")
3	#1 AND #2
4	(((ALL=(mice)) OR ALL=(mouse)) OR ALL=(rats)) OR ALL=(rat)
5	Final search query: #3 NOT #4

Trial registries searched for unpublished or ongoing studies:

- ClinicalTrials.gov
- The EU clinical trials register
